# 

**Published:** 1913-07

**Authors:** 


### INDEX.

A Tribute ........................................ 535
A Tribute from Dr. Bogart............................ 535
Abstracts and Selections.................................
......38, 81, 122, 169, 221, 266, 316, 368, 415, 471, 507, 568
Acute Indigestion. By Alf Irby, M. D., Weatherford, Texas. 429
Adolescence. By Alf Irby, M. D., Weatherford, Texas...... 237
Alcohol and Gastro-Enterostomy for the Cure of Chronic Alcoholism. By J. W. Kenney, M. D., San Antonio, Texas.. 333
Better Babies. By Theo. Y. Hull, M. D., San Antonio, Texas ........................................ 443
Cancer of the Uterus. By H. C. Crowell, M. D., Kansas City, Mo........................................ 357
Chronic Intestinal Stasis. By William Seaman Bainbridge, M. D., New York Polyclinic Medical School........... 319
Cholecystitis and Some of Its Sequelae. By A. F. Sampson, M. D., San Francisco, Cal.......................... 1
Books and Magazines...........84, 182, 280, 324, 378, 426, 474
Beri-Beri. By Paul G. Worley, M. D., Cincinnati......... 510
Diet and Prolongation of Life. By John C. Warbrick, M. D., Chicago, Ill........................................ 6
Dr. Grace's Tribute.................................... 529
Editorial Department ...................................
......33, 76, 116, 163, 216, 263, 313, 364, 412, 462, 501, 558
Editorial Notes, News and Miscellany.....................
.......36, 80, 120, 167, 219, 264, 315, 365, 412, 467, 505, 566
Eugenics Department ...................................
........13, 56, 97, 145, 199, 245, 293, 340, 393, 443, 487, 539
Eugenic Parenthood and Its Relation to Christian and Social Ethics. By M. E. Teats, M. D., Chicago................ 400
Experimental Implantation of the Generative Glands. By G. Frank Lydston, M. D., Chicago......................... 471
Expressions of Condolence............................... 536
Eyesight. Henry Copley Greene.......................... 453
Headaches. By Ernest H. Koch, M. D., Louisville, Ky.... 484
Health Supervision in Schools. By L. W. Sackett, Ph. D., Austin, Texas ...................................... 65
Importance of the Eugenic Movement and Its Relation to Social Hygiene. By Llewellyn F. Barker, Baltimore....... 145
Is the Young Man Safe? By Theo. Y. Hull, M. D., San Antonio, Texas ........................................ 99
John Allen Wyeth, M. D., LL. D., the Surgeon, the Builder, the Teacher. By John Wesley Long, M. D., Greensboro, N. C.............................................. 47

Medical Societies. By Roy W. Dunlap, M. D., Palestine, Texas .... 243
Obstetric Experience in Twenty-nine Years. By R. G. Loyd, M. D., Royse City, Texas.... 432
Observations on Pruritus Ani. By Dwight H. Murray, M. D., Syracuse, N. Y.... 422
Occupational Neuroses. By Dr. T. A. Williams, Washington, D. C.... 434
Patent Medicine. By E. H. Golar, Chemist, Food and Drug Department, State of Texas.... 111
Problems of Social Hygiene. By Theo. Y. Hull, M. D., San Antonio, Texas .... 199
Pure Food and Efficient Living. By J. S. Abbott, Texas Pure Food and Drug Commissioner.... 255
Pacifiers. By Marianna Wheeler, New York.... 457
Publisher's Department..43, 88, 185, 228, 281, 329, 428, 476, 576
Phenol-Petrolatum Treatment for Tuberculosis. By Stanley Sevier Warren, M. D., San Angelo, Texas.... 93
Poisoned by Food. Bulletin Texas State Board of Health.. 458
Radium and Cancer. By R. H. L. Bibb, M. D., Corpus Christi, Texas .... 501
Reminiscences of Hawaii. By John C. Warrick, M. D., Chicago .... 53
Relief Bill for Clean Medical Practice. By G. Henri Bogart, M. D., Paris, Ill.... 482
Results of Tonsillectomy Under Local Anesthesia. Bryan De Forest Sheedy, M. D., New York.... 38
Rupture of Uterus and Perforation of Uterus—Report of Two Cases. By B. F. Kingsley, San Antonio, Texas.... 479
Scientific Conclusions Concerning the Vice Problem. By T. D. Crothers, M. D., Hartford, Conn.... 381
Social Malady, The. By Theo. Y. Hull, M. D., San Antonio, Texas .... 245
State Aid for Needy Mothers. By Theo. Y. Hull, M. D., San Antonio, Texas .... 340
Tragedy of Womanhood. By G. Henri Bogart, M. D., Paris, Ill.... 70
Tribute to Marcellus E. Kleberg. By A. W. Fly, M. D., Galveston, Texas .... 376
Whom the Gods Would Destroy, They First Make Mad. By G. Frank Lydston, M. D., Chicago.... 266
Working Program for the Extinction of the Defective Delinquent. By Hastings H. Hart of the Russell Sage Foundation .... 17
Woman's Department ....
......27, 65, 111, 155, 209, 255, 303, 353, 399, 453, 495, 549

### The Woman's Department

**CONDUCTED BY MRS. F. E. DANIEL**

COLLABORATORS

Dr. J. S. Abbott, Dairy and Food Commissioner of Texas; Miss Eleanor Brackenridge, President of the Equal Franchise Society, San Antonio; Dr. G. Henri Bogart, Associate Editor, Utah Medical Journal, Paris, Ill.; Mrs. Isabella Charles Davis, Second Vice President National Order of "King's Daughters," New Jersey; Mrs. Jno. S. Turner, Recording Secretary, Texas Congress of Mothers; Dr. Marvin B, Grace, Seguin; Dr. Margaret Holliday, Women's Physician, University of Texas, Austin; Dr. John S. Turner, President State Medical Association; Dr. Ralph Steiner, State Health Officer; Dr. L. H. Sackett, Instructor in the Philosophy of Education, University of Texas; Dr. F. S. White, Supt. S. W. Texas Lunatic Asylum, San Antonio, Texas; Mrs. M. C. Kersh, Dallas, Texas.

### VITAL FACTS EVERY WOMAN SHOULD KNOW.

*Teach me the way to a strong body, a pure mind, a noble character.*

"*Ignorance is not innocence.*"

